# Reactions of Standing Bipeds on Moving Platforms to Keep Their Balance May Increase the Amplitude of Oscillations of Platforms Satisfying Hooke’s Law

**DOI:** 10.1371/journal.pone.0157675

**Published:** 2016-06-15

**Authors:** Guillermo H. Goldsztein

**Affiliations:** School of Mathematics, Georgia Institute of Technology, Atlanta, Georgia, United States of America; Universidad Miguel Hernandez de Elche, SPAIN

## Abstract

Consider a person standing on a platform that oscillates laterally, i.e. to the right and left of the person. Assume the platform satisfies Hooke’s law. As the platform moves, the person reacts and moves its body attempting to keep its balance. We develop a simple model to study this phenomenon and show that the person, while attempting to keep its balance, may do positive work on the platform and increase the amplitude of its oscillations. The studies in this article are motivated by the oscillations in pedestrian bridges that are sometimes observed when large crowds cross them.

## Introduction

Consider a person walking in a straight direction. The sagittal plane refers to the plane that contains the direction perpendicular to the ground and the direction toward the person is walking. As the person walks, its center of mass oscillates, both in the vertical direction (perpendicular to the ground) and the lateral direction (perpendicular to the sagittal plane). While small, these lateral oscillations are the cause of some observed, undesired and unexpected motions of pedestrian bridges when too crowded. The physics behind the wobbling of these bridges is understood to some degree. The following is believed:

Since the center of mass of each pedestrian oscillates laterally as the pedestrian walks over the bridge, the bridge is exerting lateral forces on the pedestrians, and thus, the action-reaction principle implies the pedestrians exert lateral forces on the bridge. The forces each pedestrian exerts on the bridge are oscillating and close to periodic.

Assume *N* pedestrians are crossing the bridge. They all walk with similar frequencies but not in phase and thus, the forces the pedestrians apply on the bridge partially cancel. However, the Central Limit Theorem of probability asserts that all those forces do not add up to zero. Instead, they add up to a force that would result from having of the order of $\sqrt{N}$ pedestrians walk in phase, i.e. a force of the order of magnitude $\sqrt{N}$ times the force due to one pedestrian.

When *N* is large large enough, and the frequency of the walkers is close enough to the natural frequency of the bridge, the force described in the last paragraph is large enough to cause the bridge to begin to wobble.

As the bridge wobbles, the pedestrians react in an attempt to keep their balance. As a consequence of their response, the frequency of the walkers becomes closer to the natural frequency of the bridge and the pedestrians walk mostly in phase. In other words, the walk of most pedestrian is now synchronized. Thus, the forces they exert on the platform no longer partially cancel. Instead, these forces add up to a net force on the bridge that is larger than when the bridge is not moving and the pedestrians walk out of phase. Moreover, to try to keep their balance, not only the pedestrians walk in synchrony, but the amplitude of the oscillations of their center of mass increases because their steps are wider. As a consequence of these changes in the pedestrians gait, the amplitude of the oscillations of the bridge increases even further.

We refer the reader to [1–7] for more detail discussions on the wobbling pedestrian bridges.

Synchronization of oscillators on moving platforms also occurs in other contexts. Examples include the famous Huygens’ system of pendula [8–10] and the synchronization of metronomes [11, 12]. Thus, these mentioned works are relevant to the study of the dynamics of pedestrians-bridges systems. In this work, however, as explained below in this introduction, we will not consider collections of pedestrians, only a single standing biped on a moving platform.

The above discussion on the wobbling of pedestrian bridges raises some questions. In particular: *How do pedestrians adjust their gait to keep their balance when they walk on a platform that is moving laterally?* We do not attempt to answer this question in this article. Instead, we study the following simpler, but still challenging and relevant problem.

Assume a person is standing on an oscillating platform that satisfies Hooke’s law. The person keeps its feet in the same position, somewhat wide apart, on the platform at all times. However, the person is free to bend its knees and move its trunk and arms as necessary to keep its balance. *What is the dynamics of the person-platform system?* In particular: *Does the amplitude of the oscillations of the platform increase due to the person-platform interactions?*

In this article, we introduce a model to argue on possible answers to the two questions of the last paragraph. Our analysis shows a possible feedback mechanism that leads to an increase in the amplitude of the oscillations of the platform. Our results suggest experiments to further understand this phenomenon and they also provide guidelines to undertake the more complex study of the pedestrians-bridge interaction that motivates this article.

The study of balance and stability of standing bipeds has captured the attention of many researchers [13–21]. Most investigations study the role of the ankles and hips in keeping the balance of standing persons on non-moving platforms [22–25]. As the up right equilibrium of an inverted pendulum, standing is inherently unstable. While standing, even if the person is not performing any other task and is trying to stay still on a non-moving floor, its center of mass is in constant motion. This motion is of small amplitude, sometimes unnoticeable to the naked eye. Nevertheless, this motion is there and the person avoids falling and keeps its balance by using its joints, ankles, hips and knees, to apply forces and torques [26–33]. A large amount of work in the literature is concerned with the understanding of these unconscious “strategies” used by persons to keep balance while standing. While most of the works encountered in the literature focus on the motion in the sagittal plane and on persons standing on non-moving floors [34–40], motions of the center of mass in all directions [41–45], not just restricted to the sagittal plane, and persons standing on moving platforms [46–49], have also been considered.

Experimental studies on the motions of humans standing on moving platforms were studied in [50–52], but these platforms did not move laterally, they moved in the direction the persons were facing. The need to take a step to keep balance on an accelerating platform was studied in [53]. We mention that lateral balance and control during walking was studied in [40, 54–58] and during running in [59].

This article is organized as follows. We first describe the basics of our model. We then analyze the case when there is no relative motion between the center of mass of the person and the platform. We next consider platforms that satisfy Hooke’s law, model the reactions of persons to changes in the accelerations of platforms, derive the equations governing the dynamics of a person-platform system, and analyze those equations. We finish the article with a small discussion.

## The model

We model the person as a point mass *m* and two straight segments. The mass *m* models the center of mass of the person and the two segments its legs. Both legs have the mass *m* as a common end point. The other end points of the legs are the feet. To model the ability of the person to bend its knees and to move its center of mass in general, the legs are allowed to change their length over time individually. We will refer to our model as the *model person* or *model biped*.

**Fig 1:**
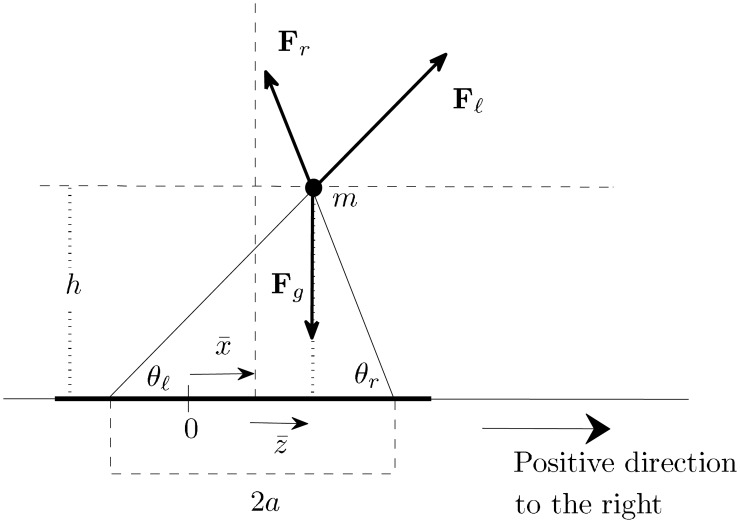
Model person and forces acting on *m*. The thick horizontal line is the platform. 0 denotes the origin of the non-moving reference outside the platform. $\bar{x}$ is the position of the midpoint between the feet in this reference frame. $\bar{x}$ and $\bar{z}$ change with time.

The model person is illustrated in Fig 1. The mass *m* is the dark solid circle. The legs are the solid thin lines. The horizontal solid line is the platform where the person stands. The feet are somewhat wide apart. The distance between both feet is 2*a*. The vertical dashed line is the line perpendicular to the platform that contains the midpoint between the feet. The distance from this line to the mass *m* is $|\bar{z}|$. We take $\bar{z}>0$ if *m* is to the right of the mentioned dashed line and $\bar{z}\leq 0$ otherwise. In the example of Fig 1, $\bar{z}>0$. The angles that the right and left legs make with the platform are *θ*_*r*_ and *θ_ℓ_* respectively (see Fig 1). *h* is the height of the model person, i.e. the distance from the mass to the platform. Note that we are modeling a two-dimensional person that is looking into the page. The platform is one-dimensional, it is a segment.

We denote the time by $\bar{t}$. The platform may move only in the horizontal direction, i.e. in the direction parallel to itself. Thus, it is only necessary to keep track of the position of only one point in the platform, say the midpoint between the feet. We denote by $\bar{x}$ the position of that point. $\bar{x}$ increases as the platform moves to the right. Note that $\bar{x}$, $\bar{z}$, *θ*_*r*_ and *θ_ℓ_* are all functions of time $\bar{t}$.

In summary, $\bar{x}$ is the position of the midpoint between the feet in a non-moving reference frame outside the platform, $\bar{z}$ is the lateral displacement of the mass *m* relative to the midpoint between the feet and thus, $\bar{x}+\bar{z}$ is the horizontal component of the position of the mass *m* in the non-moving reference frame outside the platform (see Fig 1).

**Assumption 1** *Each foot remains on the same point of the platform at all times, i.e. the feet move with the platform, but not relative to the platform*.

**Assumption 2** *We assume that the mass does not move beyond the feet, i.e*. $|\bar{z}|<a$ *or equivalently* 0 *< θ_ℓ_ < π/2 and* 0 *< θ_r_ < π/2 for all* $\bar{t}$.

Assumption 2 is just a modeling assumption. It is well understood that the center of mass could be temporarily beyond the feet without the person losing balance. In the context of our model person, as we explain later in this section, each leg exerts a force on the center of mass that is parallel to that leg and pointing away from its foot. Thus, if the center of mass is beyond the right foot, the horizontal component of both forces from the legs will be pointing to the right. A quick thought would lead to believe that the mass will move toward the right further and the model person lose its balance. However, the trajectory of a particle depends not only on the forces acting on the particle, but also on its initial velocity. In our context, if the center of mass is beyond the right foot but its *initial* velocity is to the left, the center of mass will decelerate but continue moving to the left for some period of time. Thus, if the initial speed was large enough or the center of mass was only slightly to the right of the right foot, the center of mass may return to a position between both feet. More detail explanations and analysis on this effect known as *the condition for dynamic stability* are given in the work [14]. Later in the text, we return to a discussion justifying Assumption 2.

Note that *h*, $\bar{z}$, *θ_ℓ_* and *θ*_*r*_ are related to each other through the following two equations

$$\begin{array}{c} \tan\theta_{\ell }=\frac{h}{a+\bar{z}}\;\text{and}\;\tan\theta_{r}=\frac{h}{a-\bar{z}}. \end{array}$$ (1)

**Assumption 3** *We assume that h, the distance from the mass to the platform, is constant (independent of* $\bar{t}$).

Assuming that *h* is independent of time is not fundamental or necessary, but does slightly simplify the calculations, and nothing is gained by allowing *h* to change with $\bar{t}$.

The forces acting on *m* are the force of gravity, **F**_*g*_, the force due to the left leg, **F**_*ℓ*_, and the force due to the right leg **F**_*r*_. The magnitude of **F**_*g*_ is *mg*, where *g* is the acceleration due to gravity. The legs are mass-less and thus, the net force and net torque on each leg are zero, which implies that **F**_*ℓ*_ and **F**_*r*_ are parallel to the left and right leg respectively.

The feet are not glued to the ground. It is the force of friction that keeps them in place. This is only possible if **F**_*ℓ*_ and **F**_*r*_ point from the mass in the direction opposite to the left and right foot respectively. Otherwise, if for example **F**_*r*_ points from the mass to the right foot, the action-reaction principle would imply that the right foot would feel a force pointing toward the center of mass. This force would lift the foot of the ground and **F**_*r*_ would become **0** immediately. As it will be explained later in the text, if **F**_*ℓ*_ or **F**_*r*_ become **0**, our model predicts the model person losses balance at that point in time. The magnitudes of **F**_*ℓ*_ and **F**_*r*_ will be denoted by *F_ℓ_* and *F*_*r*_ respectively.

We denote by $r_{m}=r_{m}\left(\bar{t}\right)$ the position of the mass *m* at time $\bar{t}$. According to the above discussions and Newton’s third law

$$\begin{array}{c} mr_{m}^{\prime \prime }=F_{\ell }+F_{r}+F_{g}, \end{array}$$ (2)

where primes denote derivatives with respect to $\bar{t}$.

The acceleration of the mass *m* in the horizontal direction is ${\bar{x}}^{\prime \prime }+{\bar{z}}^{\prime \prime }$ (positive to the right). On the other hand, since *h* remains constant, the acceleration of *m* in the vertical direction is 0. Thus, the vector Eq (2) can be written as the following two scalar equations:

$$m\left({\bar{x}}^{\prime \prime }+{\bar{z}}^{\prime \prime }\right)=F_{l}\text{cos}\theta_{l}-F_{r}\text{cos}\theta_{r}$$ (3)

$$0=F_{l}\text{sin}\theta_{l}+F_{r}\text{sin}\theta_{r}-mg.$$ (4)

The magnitude of the forces are non-negative and thus, we have the following restrictions:

$$\begin{array}{c} F_{\ell }\geq 0\;\text{and}\;F_{r}\geq 0. \end{array}$$ (5)

## No relative motion between the mass and platform

In this section, we consider the case when there is no relative motion between the mass and the platform. That means $\bar{z}$ is constant, independent of time. Thus, ${\bar{z}}^{\prime \prime }=0$. In other words, the model person keeps the length of its legs constant. Note that the angles *θ_ℓ_* and *θ*_*r*_ are also independent of time in this case.

Under these conditions, we can use Eqs (3) and (4) to solve for *F_ℓ_* and *F*_*r*_

$$F_{l}=m\frac{\left({\bar{x}}^{\prime \prime }\text{sin}\theta_{r}+g\text{cos}\theta_{r}\right)}{\text{sin}\left(\theta_{l}+\theta_{r}\right)}$$ (6)

$$F_{r}=m\frac{\left(-{\bar{x}}^{\prime \prime }\text{sin}\theta_{l}+g\text{cos}\theta_{l}\right)}{\text{sin}\left(\theta_{l}+\theta_{r}\right)}.$$ (7)

Since 0 < *θ_ℓ_* < *π*/2 and 0 < *θ*_*r*_ < *π*/2, the last two equations imply that *F_ℓ_* > 0 and *F*_*r*_ > 0 if ${\bar{x}}^{\prime \prime }=0$. Note that both *F_ℓ_* and *F*_*r*_ are linear functions of ${\bar{x}}^{\prime \prime }$ with positive and negative slope respectively (see Eqs (6) and (7)). The zeros of *F_ℓ_* and *F*_*r*_ are ${\bar{x}}^{\prime \prime }=-g\cot\theta_{r}$ and ${\bar{x}}^{\prime \prime }=g\cot\theta_{\ell }$ respectively. Thus, the constrains of Eq (5) translate into the following constrains on ${\bar{x}}^{\prime \prime }$:

$$\begin{array}{c} -g\cot\theta_{r}\leq {\bar{x}}^{\prime \prime }\leq g\cot\theta_{\ell }. \end{array}$$ (8)

The physical meaning of the constrains of Eq (8) is the following. When ${\bar{x}}^{\prime \prime }=g\cot\theta_{\ell }$, we have that *F*_*r*_ = 0 and *F_ℓ_* = *mg*/sin *θ_ℓ_*. In particular, the vertical component of **F**_*ℓ*_ is *mg*. If ${\bar{x}}^{\prime \prime }$ increases any further, *F_ℓ_* would also have to increase for the mass to move with the platform. That would mean that the vertical component of **F**_*ℓ*_ would be larger than *mg* and thus, since *F*_*r*_ can not become negative, the mass would accelerate upward and the feet would leave the ground, which would immediately set **F**_*ℓ*_ = **0**. This is impossible.

Instead, what happens is that the mass can not keep up with the platform. If the length of the left leg remains constant, once ${\bar{x}}^{\prime \prime }$ increases beyond the critical value of *g* cot *θ*_ℓ_, the right foot loses contact with the ground, and the left leg and the mass behave like an inverted pendulum, with the mass moving counterclockwise, i.e. to the left relative to the platform. Shortly after the mass falls to the ground. In short, the model person losses its balance and falls.

From Eqs (6) and (7) we see that, if the platform acceleration is positive, ${\bar{x}}^{\prime \prime }>0$, and both angles are equal, *θ_ℓ_* = *θ*_*r*_, the model person requires *F_ℓ_* > *F*_*r*_ to keep up with the platform. The model person will feel that its left leg is exerting a force with larger magnitude than its right leg. This is an undesirable feeling, as the model person wants to feel that both legs are exerting forces of similar magnitude, which would give the model person a better sense of stability.

The above paragraph raises the question: *Given a platform acceleration ${\bar{x}}^{\prime \prime }$, what pair of angles *θ_ℓ_* and *θ*_*r*_ make the magnitude of the forces from both legs equal?*. The answer is obtained by setting *F_ℓ_* = *F*_*r*_ and using Eqs (6) and (7). The pair of angles *θ_ℓ_* and *θ*_*r*_ that make *F*_ℓ_ = *F*_*r*_ satisfy ${\bar{x}}^{\prime \prime }=g\left(\cos\theta_{\ell }-\cos\theta_{r}\right)/\left(\sin\theta_{\ell }+\sin\theta_{r}\right)$ in addition to Eq (1). According to the previous paragraph, this pair of angles would give the model person the optimal sense of stability. Note that this *optimal* pair of angles satisfy *θ_ℓ_* < *θ*_*r*_ if ${\bar{x}}^{\prime \prime }>0$, and *θ_ℓ_* > *θ*_*r*_ if ${\bar{x}}^{\prime \prime }<0$. To achieve *θ_ℓ_* < *θ*_*r*_, the model person needs to move its center of mass to the right of the vertical line through the midpoint of the feet (the dashed vertical line in Fig 1) and analogously, to achieve *θ_ℓ_* > *θ*_*r*_, the model person needs to move its center of mass to the left of the mentioned line.

Given the above discussion, we expect the model person to try to keep its center of mass to the right of the vertical line through the midpoint of the feet when ${\bar{x}}^{\prime \prime }>0$ and to the left of this line when ${\bar{x}}^{\prime \prime }<0$. Note that this reaction can be also further justified from Eq (8). If ${\bar{x}}^{\prime \prime }>0$, moving the center of mass to the right lowers *θ_ℓ_*, and thus, increases *g* cot *θ_ℓ_*, making the range of positive platforms accelerations that the model person can tolerate larger.

For future reference, we summarize the lessons learned from above discussion in the next Observation.

**Observation 4**
1. *If* ${\bar{z}}^{\prime \prime }=0$, *the model person losses its balance if* ${\bar{x}}^{\prime \prime }>g\cot\theta_{\ell }$ *or* ${\bar{x}}^{\prime \prime }<-g\cot\theta_{r}$.
2. *The smaller θ_ℓ_, the larger positive platform accelerations the model person can tolerate without losing its balance*. *The smaller θ_r_, the larger, in absolute value, negative platform accelerations the model person can tolerate without losing its balance*.
3. *If the platform acceleration is positive*, ${\bar{x}}^{\prime \prime }>0$, *the model person wants to have its center of mass to the right of the vertical line through the midpoint of the feet, i.e. the model person wants* $\bar{z}>0$. *If* ${\bar{x}}^{\prime \prime }<0$, *the model person wants* $\bar{z}<0$.

While simple, the above are key observations in our modeling.

## Platforms moving according to Hooke’s law

We remind the reader that the platform is only free to move in the horizontal direction. Third Newton’s law (force = mass x acceleration) says that the horizontal component of the forces of the legs on the mass *m* add up to $m({\bar{x}}^{\prime \prime }+{\bar{z}}^{\prime \prime })$ because ${\bar{x}}^{\prime \prime }+{\bar{z}}^{\prime \prime }$ is the horizontal component of the acceleration of the mass *m*. Since the legs of the model person are mass-less, the total force on the legs is zero. Thus, due to the action-reaction principle, the legs exert on the platform a total force whose horizontal component is $-m({\bar{x}}^{\prime \prime }+{\bar{z}}^{\prime \prime })$.

Here and in the rest of this article, we assume that the platform follows Hooke’s law and that $\bar{x}=0$ corresponds to its equilibrium position. Thus, denoting by *M* the mass of the platform, and according to the discussion of the last paragraph, the equation describing the motion of the platform is

$$\begin{array}{c} M{\bar{x}}^{\prime \prime }=-\kappa \bar{x}-m\left({\bar{x}}^{\prime \prime }+{\bar{z}}^{\prime \prime }\right), \end{array}$$ (9)

where *κ* is the stiffness constant of the platform.

We have neglected dumping. This is a simplifying modeling assumption. All real systems contain some dumping. When forced at its natural frequency, the amplitude of a linear oscillator is limited by its dumping. If dumping is completely neglected, the amplitude is predicted to grow indefinitely. This will be the case in our analysis. Thus, by neglecting dumping, we will be not be able to predict the amplitude of the platform at which the system settles, assuming it settles before the model person loses balance. Nevertheless, we elected to neglect dumping to simplify the analysis and to isolate the effect of our interest in this article, namely, how the reactions of the model person to keep its balance affect the dynamics of the platform. Future more comprehensive studies, where more than one individual is considered and they may be walking, will certainly have to include dumping in the analysis.

We introduce the following dimensionless variables *x*, *z*, *t* by

$$\begin{array}{c} \bar{x}=ax,\;\;\bar{z}=az,\;\;\;\text{and}\;\;\;\bar{t}=\sqrt{\frac{M+m}{\kappa }}t, \end{array}$$ (10)

and the dimensionless parameter *ε* = *m*/(*M* + *m*), which we assume small

$$\begin{array}{c} \varepsilon =\frac{m}{M+m}\ll 1. \end{array}$$ (11)

Eq (9) becomes

$$\begin{array}{c} \ddot{x}=-x-\varepsilon \ddot{z}, \end{array}$$ (12)

where dots denote derivatives with respect to *t*.

If *ε* = 0, the general solution of Eq (12) is *x* = *A* sin(*t* − *ϕ*), where *A* and *ϕ* are constants, which is a periodic function of *t* with period 2*π*. In this case, we expect the reactions of the model person to also be periodic with period 2*π*. In other words, if *ε* = 0, we expect that any reasonable rules on how the model person reacts to the motion of the platform will lead to a solution *z*(*t*) that is a periodic function of *t* with period 2*π*. However, *ε* ≠ 0. Nevertheless, *ε* is small, so we expect the dynamics of the platform to not differ much from the *ε* = 0 case. In fact, the reader experienced in two-time scales asymptotic techniques may guess at this point that *x* will asymptotically be of the form *A* sin(*t* − *ϕ*), where *A* and *ϕ* are no longer constants, but change *slowly* with *t*. Similarly, we expect the reactions of the model person to lead to a solution *z*(*t*) that is *close* to a periodic function of *t*, where the precise meaning of *close* in this context is given in the next assumption.

**Assumption 5** *Given that the platform satisfies Hooke’s law, we assume that the reaction rules lead to an asymptotic approximation of z(t) of the form*

$$\begin{array}{c} z\left(t\right)\approx z_{0}\left(t,\varepsilon t\right)\;\;\;\;when\;\;\;\;\varepsilon \ll 1, \end{array}$$ (13)

*where z_0_ = z_0_(t, τ) is a function of two variables that is* 2*π-periodic on t*.

Note that the asymptotic approximation of *z* is obtained by replacing *τ* by *εt* in the second argument of *z*_0_.

Whether or not Assumption 5 is satisfied, depends on the reaction rules adopted by the model. This assumption will certainly be satisfied in our example of next section, and it should be satisfied by any reasonable set of rules.

The function *z*_0_ in Eq (13) depends on the particular rules adopted to model the reactions of the model person to the dynamics of the platform. An example in detail is given in the next section. In this section, we will proceed in generality so our results are available to be applied by future models.

Using a standard two-time scales asymptotic analysis, in the Appendix we obtain the asymptotic form of the long time dynamics of the platform-model person system. Our results are: Let *A* = *A*(*τ*) and *ϕ* = *ϕ*(*τ*) be the solutions of the following system of first order differential equations

$$\frac{dA}{d\tau }\left(\tau \right)=\frac{\left(-1\right)}{2\pi }\int_{0}^{2\pi }\frac{\partial^{2}z_{0}}{\partial t^{2}}\left(t,\tau \right)\text{cos}\left(t-\phi \left(\tau \right)\right)dt$$ (14)

$$\frac{d\phi }{d\tau }\left(\tau \right)=\frac{\left(-1\right)}{2\pi A\left(\tau \right)}\int_{0}^{2\pi }\frac{\partial^{2}z_{0}}{\partial t^{2}}\left(t,\tau \right)\text{sin}\left(t-\phi \left(\tau \right)\right)dt,$$ (15)

subjected to the initial conditions

$$\begin{array}{c} -A\left(0\right)\sin\left(\phi \left(0\right)\right)=x\left(0\right),\;A\left(0\right)\cos\left(\phi \left(0\right)\right)=\dot{x}\left(0\right), \end{array}$$ (16)

where *A*(0) ≥ 0. In the appendix A we show that

$$\begin{array}{c} x(t)\approx A(\varepsilon t)\sin(t-\phi (\varepsilon t))\;\;\;\;\text{in}\;\text{the}\;\text{regime}\;\;\;\;\varepsilon \ll 1. \end{array}$$ (17)

Before moving any further and solving the set of Eqs (14) to (17), we need to describe the motion of the center of mass of the model person relative to the platform, i.e. we need the rules that determine the dynamics of *z*(*t*).

## Modeling the reaction of the model person to the motion of the platform

There are several options to model the reactions of the model person to the motion of the platform. In fact, different persons may react differently, and even the same person may react differently at different times. For example, if the platform is oscillating, the person’s memory or experience is likely to lead to somewhat different reactions after each oscillation, improving its strategy to keep its balance.

In this article we will select a class of *reaction rules* described below that is motivated by Observation 4. We hope our analysis will motivate experiments to test how realistic our rules are, and improve on them.

While we will study only one class of reaction rules, our analysis can be easily adapted to study other types reactions rules, that will ideally result from measurements from experiments in the future. So we do not regard the conclusions we will obtain from our model as the final word, but rather as an opening for further studies.

Note that *z* = 1 corresponds to having the mass *m* directly above the right foot, i.e. *θ*_*r*_ = *π*/2. But according to our assumptions this never happens. We have assumed that $|\bar{z}|<a$ at all times. In other words, we have the restriction |*z*|<1.

According to our discussion which was summarized in Observation 4, a reasonable strategy the model person may adopt is that it moves its center of mass to its right, *z* > 0, each time the acceleration of the platform is positive, $\ddot{x}>0$, and viceversa, it moves its center of mass to its left, *z* < 0, each time the acceleration of the platform is negative, $\ddot{x}<0$.

There are many possible rules compatible with this strategy. For simplicity and to be concrete, we assume there is a number *λ* such that 0 < *λ* < 1 and that the model person moves its center of mass to *z* = *λ* when it feels positive platform acceleration, $\ddot{x}>0$, and viceversa, the model person moves its center of mass to *z* = −*λ* when it feels a negative platform acceleration, $\ddot{x}<0$.

Our next step is to be more precise and prescribe a rule or modeling assumption on how the model person moves its center of mass from *z* = *λ* to *z* = −*λ* as it senses that $\ddot{x}$ becomes negative. That rule is given in Assumption 6, or equivalently, Eq (20). There is nothing special about that rule other that it is in accordance to the discussion of this section and its functional form is simple enough to allow us to carry out calculations in detail.

We remind the reader of the approximation of Eqs (14), (15), (16) and (17), from where we get

$$\begin{array}{c} \ddot{x}\left(t\right)\approx -A\sin\left(t-\phi \right), \end{array}$$ (18)

where *A* = *A*(*εt*) and *ϕ* = *ϕ*(*εt*) and again, the above equation means that −*A* sin(*t* − *ϕ*) is the asymptotic approximation of $\ddot{x}\left(t\right)$ in the regime *ε* ≪ 1. We do not explicitly show in Eq (18) that *A* and *ϕ* are evaluated in *εt* because in the subsequent calculations, we will treat them as constants, which is asymptotically correct since *ε* ≪ 1. This last equation and the discussion preceding it are our motivation for our next modeling assumption.

**Assumption 6** *Let k be any integer*. *We define ϕ_k_ = ϕ + 2πk. We assume the response of the model person to the platform accelerations given by* Eq (18) *results in a dynamics of z(t) that in the time interval* [*ϕ_k_, ϕ*_k+1_] *is determined by the following: z(ϕ_k_) = λ;* $\dot{z}\left(\phi_{k}\right)=0$; *and that there exists α* > 0 *and T* > 0 *related by αT*^2^ = 2*λ and satisfying the constrain T* ≤ *π*/2 *such that*

$$\ddot{z}\left(t\right)=\{\begin{array}{ll} -\alpha & if\;\phi_{k}<t<\phi_{k}+T\\ \alpha & if\;\phi_{k}+T<t<\phi_{k}+2T\\ 0 & if\;\phi_{k}+2T<t<\phi_{k}+\pi \\ \alpha & if\;\phi_{k}+\pi <t<\phi_{k}+\pi +T\\ -\alpha & if\;\phi_{k}+\pi +T<t<\phi_{k}+\pi +2T\\ 0 & if\;\phi_{k}+\pi +2T<t<\phi_{k}+2\pi . \end{array}$$ (19)

Note that $\ddot{z}\left(t\right)$ is well defined because *T* ≤ *π*/2. Note also that Eq (19) together with the conditions *z*(*ϕ*_*k*_) = *λ* and $\dot{z}\left(\phi_{k}\right)=0$ can be integrated at once to give the following explicit formula for *z*(*t*)

$$\begin{array}{c} z\left(t\right)=\left\{\begin{array}{cc} \lambda -\frac{\alpha }{2}{\left(t-\phi_{k}\right)}^{2} & \text{if}\;\phi_{k}<t<\phi_{k}+T\\ -\alpha T\left(t-(\phi_{k}+T)\right)+\frac{\alpha }{2}{\left(t-(\phi_{k}+T)\right)}^{2} & \text{if}\;\phi_{k}+T<t<\phi_{k}+2T\\ -\lambda & \text{if}\;\phi_{k}+2T<t<\phi_{k}+\pi \\ -\lambda +\frac{\alpha }{2}{\left(t-(\phi_{k}+\pi )\right)}^{2} & \text{if}\;\phi_{k}+\pi <t<\phi_{k}+\pi +T\\ \alpha T\left(t-(\phi_{k}+\pi +T)\right)-\frac{\alpha }{2}{\left(t-(\phi_{k}+\pi +T)\right)}^{2} & \text{if}\;\phi_{k}+\pi +T<t<\phi_{k}+\pi +2T\\ \lambda & \text{if}\;\phi_{k}+\pi +2T<t<\phi_{k}+2\pi . \end{array}\right. \end{array}$$ (20)

**Fig 2:**
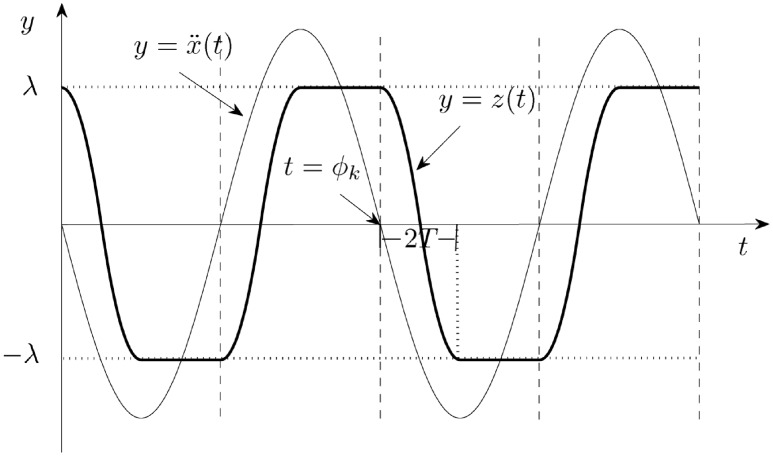
Plots of $\ddot{x}$ vs *t* (see Eq (18)) and *z* vs *t* (see Eq (20)) with parameter values *λ*/*A* = 0.7 and *T* = *π*/4.

The motivation behind the Assumption 6 is easier explained with the aid of Fig 2. The thin solid line in that figure is the plot of $\ddot{x}=-A\sin\left(t-\phi \right)$ vs *t*. The thick solid line is the plot of *z*(*t*) vs *t*. Note that, for *t* < *ϕ*_*k*_ but *t* close enough to *ϕ*_*k*_, we have that $\ddot{x}\left(t\right)>0$ and *z*(*t*) = *λ*. Note also that *t* = *ϕ*_*k*_ is a zero of $\ddot{x}$ and $\ddot{x}\left(t\right)<0$ for *ϕ*_*k*_ < *t* < *ϕ*_*k*_ + *π*. In other words, $\ddot{x}\left(t\right)$ changes from being positive to being negative as *t* increases and goes through the value *t* = *ϕ*_*k*_. Thus, in accordance to our previous discussions, and as it can be seen in Fig 2, the model person starts moving its center of mass to the left once *t* > *ϕ*_*k*_. In fact, *z*(*t*) decreases from the value of *λ* to the value of −*λ* as *t* increases from *t* = *ϕ*_*k*_ to *t* = *ϕ*_*k*_ + 2*T*. Note that 2*T* is the time the model person needs to move its center of mass relative to the platform from *z* = *λ* to *z* = −*λ*. This time is related to *α*, the absolute value of $\ddot{z}$ while *z* decreases. Easy calculations lead to the relation previously mentioned that we repeat for future reference

$$\begin{array}{c} \alpha T^{2}=2\lambda . \end{array}$$ (21)

The magnitude of the forces the legs exert on the mass *m* are limited by the fact that the height of the model person remains constant and thus, the sum of the vertical components the forces the legs exert on the mass *m* is always equal to the magnitude of the force due to gravity. As a consequence, the motion of the center of mass from *z* = *λ* to *z* = −*λ* can not be instantaneous, as an instantaneous change in position would mean infinite accelerations and thus infinite forces. In fact, in the Appendix B we obtain the following lower bound on *T*

$$\begin{array}{c} T\geq \sqrt{\frac{2\lambda h\kappa }{\left(M+m)g(1-\lambda \right)}}. \end{array}$$ (22)

Note that the period of oscillation of the bridge-person system is $T_{\text{spring}}=2\pi \sqrt{(M+m)/\kappa }$. Note also that the time an object needs to fall to the ground due to gravity from an initial height of *h* is $T_{\text{gravity}}=\sqrt{2h/g}$. Thus, the bound of Eq (22) on *T* together with the fact that *T* ≤ *π*/2, can be written as

$$\begin{array}{c} T_{b}=2\pi \sqrt{\frac{\lambda }{\left(1-\lambda \right)}}\frac{T_{\text{gravity}}}{T_{\text{spring}}}\leq T\leq \frac{\pi }{2}. \end{array}$$ (23)

Needless to say, we need *T*_*b*_ < *π*/2 so that the range of values *T* can take is not empty. Note that *h*, the height of the mass, can be interpreted as modeling the height of the hips of a person. Assuming the person spreads its legs so that its hips are at a height of about 1/2 a meter, we have that *T*_gravity_ is about 1/3 seconds and thus, π*T*_gravity_ is about 1 second. Thus, assuming a value of *λ* ≈ 1/5, we get that $2\pi \sqrt{\lambda /(1-\lambda )}T_{\text{gravity}}$ is about to 1 second. Thus, requiring *T*_*b*_ < *π*/2 is approximately equivalent to requiring *T*_gravity_ > 2/π seconds. Or roughly speaking, that the period of oscillations of the platform is about 1 second or more.

The need for this lower bound on the the period of oscillations of the platform is so that the person has enough time to move its center of mass from side to side. Note that other reaction rules may lead to different restrictions on the period of oscillations. In fact, other reaction rules may lead to restrictions on the platform accelerations, not on the period. In short, there will always be some restrictions on the motion of the platform because if the acceleration of the platform is too large, the model person will lose its balance. In the example of this section, the restriction is Eq (23) or that the period of the platform should be of the order of one second or more.

We note that the Assumption 5 is immediately satisfied with the reaction rules we have adopted that lead to Eq (20).

## Effects on the platform oscillations due to the reactions of the model person

Given our model of the dynamics of *z*, Eq (20), we can compute the effect of the reactions of the model person (in the context of our model) on the dynamics of the platform. We will assume that the amplitude of oscillations of the platform is initially small. Our main objective is to understand whether the amplitude of the oscillations will increase in time or not.

More precisely, using Eqs (19) and (21) and simple algebra, Eqs (14) and (15) become

$$\frac{dA}{d\tau }\left(\tau \right)=\frac{4\lambda }{\pi }\text{sin}T\frac{\left(1-\text{cos}T\right)}{T^{2}}$$ (24)

$$\frac{d\phi }{d\tau }\left(\tau \right)=\frac{2\lambda }{\pi A\left(\tau \right)}\frac{\left(1-2\text{cos}T+\text{cos}\left(2T\right)\right)}{T^{2}}.$$ (25)

Note that the right hand side of Eq (24) is positive, which leads to the following observation.

**Observation 7** *The amplitude of oscillations of the platform increases linearly with t. More precisely, setting* $A_{0}=A\left(0\right)=\sqrt{x^{2}\left(0\right)+{\dot{x}}^{2}\left(0\right)}$ (*see* Eq (16)), *we have*

$$\begin{array}{c} x\left(t\right)\approx \left(A_{0}+\varepsilon ft\right)\sin\left(t-\phi \left(\varepsilon t\right)\right), \end{array}$$ (26)

*where*

$$\begin{array}{c} f=\frac{4\lambda }{\pi }\sin T\frac{\left(1-\cos T\right)}{T^{2}} \end{array}$$ (27)

*where ε is as defined in* Eq (11).

Note that *f* = *f*(*T*) is non-negative in the interval 0 ≤ *T* ≤ *π*/2, and the only zero of *f* in the interval 0 ≤ *T* ≤ *π*/2 is *T* = 0. We know form the inequality of Eq (23) that *T* can not be arbitrarily small. In fact, *T* ≥ *T*_*b*_, where *T*_*b*_ was defined in Eq (23). Thus, studying the parameter regime *T* ≪ 1 makes sense only if *T*_*b*_ ≪ 1. Assuming that this is the case, Eqs (26) and (27) reduce to

$$\begin{array}{c} x\left(t\right)=\left(A_{0}+\frac{2}{\pi }\lambda T\varepsilon t\right)\sin\left(t-\phi \left(\varepsilon t\right)\right),\;\text{if}\;T\ll 1. \end{array}$$ (28)

Note that the faster the person can move its center of mass from side to side, the slower the rate at which the amplitude of the platform oscillations increases. If this motion of the center of mass were instantaneous, the person would not affect the dynamics of the platform. But we know this is impossible and thus, at least within our model, the amplitude of the platform oscillations always increases.

## Discussion

In this article, we studied how a person standing on an oscillating platform that satisfies Hooke’s law moves its body, and thus its center of mass, to keep its balance, and how those movements of its center of mass affect the motion of the platform. We introduced and analyzed a simple mathematical model. We showed that, within our model, the motions of the model person lead to an increase of the amplitude of oscillations of the platform in time, an undesirable side effect from the point of view of the person.

A critical part of our modeling consists in prescribing *rules* on how the model person reacts to the dynamics of the platform. We motivated and proposed a set of *rules*, but many other rules are possible. Thus, while we believe the rules we proposed are reasonable, the conclusion obtained with our rules, namely that the amplitude of the platform oscillations increases with time, should be considered as a *possibility* in real systems, and not as *definite final word*. The rules proposed here also serve as an example to illustrate how the analysis presented in this article can be used, since our analysis can be easily adapted to different rules. The rules used in this study did not result from experimental measurements, but rather from thoughtful consideration and speculation on how we expect persons to react when standing on moving platforms.

We accomplished our two goals, namely, to introduce a method to analyze the dynamics of the platform-person system, and to show that, as a result of the platform-person interactions, an increase of the amplitude of the oscillations of the platform with time is *possible*, even though that is not the intention of the person. In fact, the intention of the person is to keep its balance and thus, it would prefer that the platform does not move.

With this article, we hope to motivate further research in two directions. On the theoretical side, we plan to extend the analysis presented here to persons walking on moving platforms instead of just standing, and to study the interactions of platforms with several persons, instead of just one. After all, the wobbling of pedestrian bridges is the phenomenon that motivated this article.

On the experimental side, we hope experimentalists will design and perform experiments to better understand how standing and walking persons react to the lateral motion of the surface where the persons are standing or walking. We propose the following experiments, admitting that due to our lack of experience with experiments, we can not asses how feasible or difficult these experiments are to carry out.

*Experiment 1*: Simply have a person standing on a platform that is moving laterally sinusoidally. Instruct the person to stand still, not bend its knees, ankles or any of its joints or arms. Measure the force exerted by each leg on the platform as a function of time, and compare those measurements with the predictions of our theory, namely, Eqs (6) and (7). This experiment would test how adequate in this context is to use a mass attached to two mass-less legs as a model of a person.

*Experiment 2*: Use a platform that can move laterally nearly following Hooke’s law, i.e. with very small dumping. Have a person standing on that platform. The mass of the platform should be considerably larger than the mass of the person if possible. Instruct the person to keep its feet in place, but otherwise he is allow to move to keep its balance. Give an initial displacement from equilibrium to the platform and study the evolution of the system. Keep track of the position, as a function of time, of both the platform and the center of mass of the individual. Obtaining the position as a function of time of the center of mass of the person may have to be done indirectly by first measuring the forces the feet exert on the platform as a function of time and then using Newton’s law. Observe if the amplitude of oscillation of the platform increases with time. Having the trajectory of the platform and the center of mass means having *x*(*t*) and *z*(*t*). Our general theory can then be tested, namely Eqs (14) to (17). It would also be interested to measure how much the height of the center of mass of the person changes with time, as we have neglected this change. The particular reaction rule used in this article could also be tested with the measurements of this experiment. One could try to come up with better reactions rule given the experimental measurements. Finally, repeat the experiment with different individuals to understand if there are general strategies used by all individuals, or if the reaction rules vary a lot from person to person.

## Appendix

### Appendix A

We will solve Eq (12) asymptotically, in the regime of Eq (11), with the use standard two-time scales techniques. We refer the reader to the book [60] for details, but briefly, without describing all the details and motivations, the steps to follow are:

Introduce a second independent variable *τ*. Regard *t* and *τ* as independent from each other. Assume *x* and *z* are functions of both *t* and *τ*. Replace time derivatives in Eq (12) as follows:

$$\begin{array}{c} \frac{d}{dt}=\frac{\partial }{\partial t}+\varepsilon \frac{\partial }{\partial \tau }, \end{array}$$ (29)

where $\frac{\partial }{\partial t}$ and $\frac{\partial }{\partial \tau }$ denote the partial derivatives with respect to *t* and *τ* respectively. Eq (12) becomes

$$\begin{array}{c} \frac{\partial^{2}x}{\partial t^{2}}+2\varepsilon \frac{\partial^{2}x}{\partial t\partial \tau }+\varepsilon^{2}\frac{\partial^{2}x}{\partial \tau^{2}}=-x\\ -\varepsilon \left(\frac{\partial^{2}z}{\partial t^{2}}+2\varepsilon \frac{\partial^{2}z}{\partial t\partial \tau }+\varepsilon^{2}\frac{\partial^{2}z}{\partial \tau^{2}}\right). \end{array}$$ (30)

After neglecting everything of order *ε*^2^ in the last equation, and moving −*x* to the left hand side, we get

$$\begin{array}{c} \frac{\partial^{2}x}{\partial t^{2}}+2\varepsilon \frac{\partial^{2}x}{\partial t\partial \tau }+x=-\varepsilon \frac{\partial^{2}z}{\partial t^{2}}+O\left(\varepsilon^{2}\right). \end{array}$$ (31)

Set the anzats *x* = *x*_0_ + *εx*_1_ + *O*(*ε*^2^) and *z* = *z*_0_ + *εz*_1_ + *O*(*ε*^2^), where *x*_0_, *x*_1_, *z*_0_ and *z*_1_ are also functions of *t* and *τ*. Plug these anzats into Eq (31), and again neglect anything of order *ε*^2^, to get

$$\begin{array}{c} \frac{\partial^{2}x_{0}}{\partial t^{2}}+\varepsilon \frac{\partial^{2}x_{1}}{\partial t^{2}}+2\varepsilon \frac{\partial^{2}x_{0}}{\partial t\partial \tau }+x_{0}+\varepsilon x_{1}=-\varepsilon \frac{\partial^{2}z_{0}}{\partial t^{2}}+O\left(\varepsilon^{2}\right). \end{array}$$

Collect powers of *ε* in the above equation to get the following two equations. At *O*(1)

$$\begin{array}{c} \frac{\partial^{2}x_{0}}{\partial t^{2}}+x_{0}=0 \end{array}$$ (32)

and at *O*(*ε*)

$$\begin{array}{c} \frac{\partial^{2}x_{1}}{\partial t^{2}}+2\frac{\partial^{2}x_{0}}{\partial t\partial \tau }+x_{1}=-\frac{\partial^{2}z_{0}}{\partial t^{2}}. \end{array}$$ (33)

The general solution of Eq (32) is

$$\begin{array}{c} x_{0}\left(t,\tau \right)=A\left(\tau \right)\sin\left(t-\phi \left(\tau \right)\right), \end{array}$$ (34)

where *A* = *A*(*τ*) and *ϕ* = *ϕ*(*τ*), i.e. *A* and *ϕ* are independent of *t* but they depend on *τ*.

Now plug the above expression for *x*_0_ into Eq (33) to get, after a small rearrangement of the terms

$$\begin{array}{c} \frac{\partial^{2}x_{1}}{\partial t^{2}}+x_{1}=-2\frac{\partial A}{\partial \tau }\cos\left(t-\phi \right)-2A\frac{\partial \phi }{\partial \tau }\sin\left(t-\phi \right)-\frac{\partial^{2}z_{0}}{\partial t^{2}}. \end{array}$$

Next, we require *x*_1_ to be a periodic function of *t*. Thus, we need the integral over the interval 0 < *t* < 2*π* of the right hand side times cos(*t* − *ϕ*) to be zero. We also need the integral over the interval 0 < *t* < 2*π* of the right hand side times sin(*t* − *ϕ*) to be zero. This leads to

$$\begin{array}{c} \frac{dA}{d\tau }\left(\tau \right)=-\frac{1}{2\pi }\int_{0}^{2\pi }\frac{\partial^{2}z_{0}}{\partial t^{2}}\left(t,\tau \right)\cos\left(t-\phi \left(\tau \right)\right)dt \end{array}$$

and

$$\begin{array}{c} \frac{d\phi }{d\tau }\left(\tau \right)=-\frac{1}{2\pi A(\tau )}\int_{0}^{2\pi }\frac{\partial^{2}z_{0}}{\partial t^{2}}\left(t,\tau \right)\sin\left(t-\phi \left(\tau \right)\right)dt. \end{array}$$

Once the solution is found, we set *τ* = *εt*. Note that this justifies the initial change prescribed by Eq (29) regarding the derivatives. Note also that, since *x*_1_ is periodic in *t*, *x*_1_(*t*, *εt*) may only grow slowly, i.e. may only become large for *t* ≫ 1. This justifies the *x* ≈ *x*_0_(*t*, *εt*) for large values of *t*. Thus, the validity of Eqs (13) to (17).

### Appendix B

We introduce the dimensionless force magnitudes *G_ℓ_* and *G*_*r*_ and the parameter *β* defined by the following equations

$$\begin{array}{c} F_{\ell }=mgG_{\ell },\;F_{r}=mgG_{\ell },\;\;\text{and}\;\;\beta =\frac{(M+m)g}{a\kappa }. \end{array}$$ (35)

Using Eq (10), the dimensionless version of Eqs (3) and (4) are

$$\ddot{x}+\ddot{z}=\beta \left(G_{l}\text{cos}\theta_{l}-G_{r}\text{cos}\theta_{r}\right)$$ (36)

$$0=G_{l}\text{sin}\theta_{l}+G_{r}\text{sin}\theta_{r}-1.$$ (37)

Since *G_ℓ_* and *G*_*r*_ are non-negative, from Eq (37) we get

$$G_{r}=\frac{1-G_{l}\text{sin}\theta_{l}}{\text{sin}\theta_{r}}\leq \frac{1}{\text{sin}\theta_{r}}$$ (38)

$$G_{l}=\frac{1-G_{r}\text{sin}\theta_{r}}{\text{sin}\theta_{l}}\leq \frac{1}{\text{sin}\theta_{l}}.$$ (39)

Thus, Eqs (36) and (37), and simple arguments lead to

$$\begin{array}{c} -\beta \cot\theta_{r}-\ddot{x}\leq \ddot{z}\leq \beta \cot\theta_{\ell }-\ddot{x}. \end{array}$$ (40)

Given Eqs (1) and (10) we have that

$$\begin{array}{c} \cot\theta_{r}=\frac{a}{h}\left(1-z\right). \end{array}$$ (41)

Thus, using Eq (41), the left inequality in Eq (40) becomes

$$\begin{array}{c} -\beta \frac{a}{h}\left(1-z\right)-\ddot{x}\leq \ddot{z}. \end{array}$$ (42)

Using the definition of *β* (Eq (35)), the fact that $\ddot{x}\approx -A\sin\left(t-\phi \right)$, and using Eqs (19) and (20), and evaluating the above inequality at *t* = *ϕ*^+^ (i.e taking the limit at *t* → *ϕ* but *t* > *ϕ*), we get

$$\begin{array}{c} -\frac{(M+m)g}{h\kappa }\left(1-\lambda \right)\leq -\alpha , \end{array}$$ (43)

which is equivalent to Eq (22) because 2*λ* = *αT*^2^.

## References

[1] Bocian M, Macdonald J, Burn J. Biomechanically inspired modelling of pedestrian-induced forces on laterally oscillating structures. Journal of Sound and Vibration. 2012;331(16):3914–3929. 10.1016/j.jsv.2012.03.023

[2] Bodgi J, Erlicher S, Argoul P. Lateral vibration of footbridges under crowd-loading: continuous crowd modeling approach In: Key Engineering Materials. vol. 347 Trans Tech Publ; 2007 p. 685–690.

[3] Bruno L, Venuti F, Nascé V. Pedestrian-induced torsional vibrations of suspended footbridges: Proposal and evaluation of vibration countermeasures. Engineering Structures. 2012;36:228–238. 10.1016/j.engstruct.2011.12.012

[4] Dallard P, Fitzpatrick T, Flint A, Low A, Smith RR, Willford M, et al London Millennium Bridge: pedestrian-induced lateral vibration. Journal of Bridge Engineering. 2001;6(6):412–417. 10.1061/(ASCE)1084-0702(2001)6:6(412)

[5] Ingólfsson ET, Georgakis CT, Jönsson J. Pedestrian-induced lateral vibrations of footbridges: A literature review. Engineering Structures. 2012;45:21–52. 10.1016/j.engstruct.2012.05.038

[6] Macdonald JH. Lateral excitation of bridges by balancing pedestrians. In: Proceedings of the Royal Society of London A: Mathematical, Physical and Engineering Sciences. The Royal Society; 2008. p. rspa–2008.

[7] Venuti F, Bruno L. Crowd-structure interaction in lively footbridges under synchronous lateral excitation: A literature review. Physics of Life Reviews. 2009;6(3):176–206. 10.1016/j.plrev.2009.07.001 20416851

[8] Huygens C. Christiaan Huygens’ the pendulum clock, or, Geometrical demonstrations concerning the motion of pendula as applied to clocks. Iowa State Pr; 1986.

[9] Bennett M, Schatz MF, Rockwood H, Wiesenfeld K. Huygens’s clocks. Proceedings: Mathematics, Physical and Engineering Sciences. 2002; p. 563–579.

[10] Koluda P, Perlikowski P, Czolczynski K, Kapitaniak T. Synchronization configurations of two coupled double pendula. Communications in Nonlinear Science and Numerical Simulation. 2014;19(4):977–990. 10.1016/j.cnsns.2013.08.008

[11] Pantaleone J. Synchronization of metronomes. American Journal of Physics. 2002;70(10):992–1000. 10.1119/1.1501118

[12] Ulrichs H, Mann A, Parlitz U. Synchronization and chaotic dynamics of coupled mechanical metronomes. Chaos: An Interdisciplinary Journal of Nonlinear Science. 2009;19(4):043120 10.1063/1.3266924 20059216

[13] Balasubramaniam R, Wing AM. The dynamics of standing balance. Trends in cognitive sciences. 2002;6(12):531–536. 10.1016/S1364-6613(02)02021-1 12475714

[14] Hof A, Gazendam M, Sinke W. The condition for dynamic stability. Journal of biomechanics. 2005;38(1):1–8. 10.1016/j.jbiomech.2004.03.025 15519333

[15] Hof AL. The equations of motion for a standing human reveal three mechanisms for balance. Journal of biomechanics. 2007;40(2):451–457. 10.1016/j.jbiomech.2005.12.016 16530203

[16] Hsu WL, Scholz JP, Schöner G, Jeka JJ, Kiemel T. Control and estimation of posture during quiet stance depends on multijoint coordination. Journal of neurophysiology. 2007;97(4):3024–3035. 10.1152/jn.01142.2006 17314243

[17] Riley M, Balasubramaniam R, Turvey M. Recurrence quantification analysis of postural fluctuations. Gait & posture. 1999;9(1):65–78. 10.1016/S0966-6362(98)00044-7 10575072

[18] Wang Z, Ko JH, Challis JH, Newell KM. The degrees of freedom problem in human standing posture: collective and component dynamics. PloS one. 2014;9(1):e85414 10.1371/journal.pone.0085414 24427307 PMC3888423

[19] Winter DA, Patla AE, Prince F, Ishac M, Gielo-Perczak K. Stiffness control of balance in quiet standing. Journal of neurophysiology. 1998;80(3):1211–1221. 9744933 10.1152/jn.1998.80.3.1211

[20] Winter DA, Patla AE, Ishac M, Gage WH. Motor mechanisms of balance during quiet standing. Journal of Electromyography and Kinesiology. 2003;13(1):49–56. 10.1016/S1050-6411(02)00085-8 12488086

[21] Woollacott M, Shumway-Cook A. Attention and the control of posture and gait: a review of an emerging area of research. Gait & posture. 2002;16(1):1–14. 10.1016/S0966-6362(01)00156-4 12127181

[22] Gatev P, Thomas S, Kepple T, Hallett M. Feedforward ankle strategy of balance during quiet stance in adults. The Journal of physiology. 1999;514(3):915–928. 10.1111/j.1469-7793.1999.915ad.x 9882761 PMC2269093

[23] Masani K, Popovic MR, Nakazawa K, Kouzaki M, Nozaki D. Importance of body sway velocity information in controlling ankle extensor activities during quiet stance. Journal of Neurophysiology. 2003;90(6):3774–3782. 10.1152/jn.00730.2002 12944529

[24] Park S, Horak FB, Kuo AD. Postural feedback responses scale with biomechanical constraints in human standing. Experimental Brain Research. 2004;154(4):417–427. 10.1007/s00221-003-1674-3 14618285

[25] Suzuki Y, Nomura T, Casadio M, Morasso P. Intermittent control with ankle, hip, and mixed strategies during quiet standing: a theoretical proposal based on a double inverted pendulum model. Journal of Theoretical Biology. 2012;310:55–79. 10.1016/j.jtbi.2012.06.019 22732276

[26] Bardy B, Oullier O, Bootsma RJ, Stoffregen TA. Dynamics of human postural transitions. Journal of Experimental Psychology: Human Perception and Performance. 2002;28(3):499 12075884

[27] Black FO, Wall C, Rockette HE, Kitch R. Normal subject postural sway during the Romberg test. American journal of Otolaryngology. 1982;3(5):309–318. 10.1016/S0196-0709(82)80002-1 7149143

[28] Brunt D, Andersen J, Huntsman B, Reinhert L, Thorell A, Sterling J. Postural responses to lateral perturbation in healthy subjects and ankle sprain patients. Medicine and science in sports and exercise. 1992;24(2):171–176. 1549005

[29] Carpenter MG, Frank JS, Silcher CP, Peysar GW. The influence of postural threat on the control of upright stance. Experimental Brain Research. 2001;138(2):210–218. 10.1007/s002210100681 11417462

[30] Carpenter M, Murnaghan C, Inglis J. Shifting the balance: evidence of an exploratory role for postural sway. Neuroscience. 2010;171(1):196–204. 10.1016/j.neuroscience.2010.08.030 20800663

[31] Kuo AD. An optimal state estimation model of sensory integration in human postural balance. Journal of Neural Engineering. 2005;2(3):S235. 16135887 10.1088/1741-2560/2/3/S07

[32] Kuo AD, Zajac FE. Human standing posture: multi-joint movement strategies based on biomechanical constraints. Progress in brain research. 1992;97:349–358. 10.1016/S0079-6123(08)62294-3 8234760

[33] Kuo AD, Zajac FE. A biomechanical analysis of muscle strength as a limiting factor in standing posture. Journal of Biomechanics. 1993;26:137–150. 10.1016/0021-9290(93)90085-S 8505348

[34] Bottaro A, Yasutake Y, Nomura T, Casadio M, Morasso P. Bounded stability of the quiet standing posture: an intermittent control model. Human movement science. 2008;27(3):473–495. 10.1016/j.humov.2007.11.005 18342382

[35] Gage WH, Winter DA, Frank JS, Adkin AL. Kinematic and kinetic validity of the inverted pendulum model in quiet standing. Gait & posture. 2004;19(2):124–132. 10.1016/S0966-6362(03)00037-7 15013500

[36] McCollum G, Leen TK. Form and exploration of mechanical stability limits in erect stance. Journal of Motor Behavior. 1989;21(3):225–244. 10.1080/00222895.1989.10735479 15136262

[37] Nawayseh N, Griffin MJ. Effect of frequency, magnitude and direction of translational and rotational oscillation on the postural stability of standing people. Journal of Sound and Vibration. 2006;298(3):725–754. 10.1016/j.jsv.2006.06.027

[38] Otten E. Balancing on a narrow ridge: biomechanics and control. Philosophical Transactions of the Royal Society of London B: Biological Sciences. 1999;354(1385):869–875. 10.1098/rstb.1999.0439 10382221 PMC1692595

[39] Pai YC, Patton J. Center of mass velocity-position predictions for balance control. Journal of biomechanics. 1997;30(4):347–354. 10.1016/S0021-9290(96)00165-0 9075002

[40] Winter DA. Human balance and posture control during standing and walking. Gait & posture. 1995;3(4):193–214. 10.1016/0966-6362(96)82849-9

[41] Collins JJ, De Luca CJ. Open-loop and closed-loop control of posture: a random-walk analysis of center-of-pressure trajectories. Experimental brain research. 1993;95(2):308–318. 10.1007/BF00229788 8224055

[42] Winter D, Prince F, Stergiou P, Powell C. Medial-lateral and anterior-posterior motor-responses associated with center of pressure changes in quiet standing. Neuroscience Research Communications. 1993;12(3):141–148.

[43] O’Connor SM, Kuo AD. Direction-dependent control of balance during walking and standing. Journal of Neurophysiology. 2009;102(3):1411–1419. 10.1152/jn.00131.2009 19553493 PMC2746770

[44] Rietdyk S, Patla A, Winter D, Ishac M, Little C. Balance recovery from medio-lateral perturbations of the upper body during standing. Journal of biomechanics. 1999;32(11):1149–1158. 10.1016/S0021-9290(99)00116-5 10541064

[45] Winter DA, Prince F, Frank J, Powell C, Zabjek KF. Unified theory regarding A/P and M/L balance in quiet stance. Journal of neurophysiology. 1996;75(6):2334–2343. 8793746 10.1152/jn.1996.75.6.2334

[46] Creath R, Kiemel T, Horak F, Peterka R, Jeka J. A unified view of quiet and perturbed stance: simultaneous co-existing excitable modes. Neuroscience letters. 2005;377(2):75–80. 10.1016/j.neulet.2004.11.071 15740840

[47] Henry SM, Fung J, Horak FB. Effect of stance width on multidirectional postural responses. Journal of neurophysiology. 2001;85(2):559–570. 11160493 10.1152/jn.2001.85.2.559

[48] Kuo AD. An optimal control model for analyzing human postural balance. Biomedical Engineering, IEEE Transactions on. 1995;42(1):87–101. 10.1109/10.362914 7851935

[49] Scholz J, Schöner G, Hsu W, Jeka J, Horak F, Martin V. Motor equivalent control of the center of mass in response to support surface perturbations. Experimental brain research. 2007;180(1):163–179. 10.1007/s00221-006-0848-1 17256165

[50] Ko YG, Challis JH, Newell KM. Postural coordination patterns as a function of dynamics of the support surface. Human movement science. 2001;20(6):737–764. 10.1016/S0167-9457(01)00052-5 11792438

[51] Ko YG, Challis JH, Newell KM. Learning to coordinate redundant degrees of freedom in a dynamic balance task. Human Movement Science. 2003;22(1):47–66. 10.1016/S0167-9457(02)00177-X 12623180

[52] Ko JH, Challis JH, Newell KM. Postural coordination patterns as a function of rhythmical dynamics of the surface of support. Experimental brain research. 2013;226(2):183–191. 10.1007/s00221-013-3424-5 23392472

[53] Pai YC, Maki B, Iqbal K, McIlroy W, Perry S. Thresholds for step initiation induced by support-surface translation: a dynamic center-of-mass model provides much better prediction than a static model. Journal of biomechanics. 2000;33(3):387–392. 10.1016/S0021-9290(99)00199-2 10673124

[54] Bauby CE, Kuo AD. Active control of lateral balance in human walking. Journal of biomechanics. 2000;33(11):1433–1440. 10.1016/S0021-9290(00)00101-9 10940402

[55] Donelan JM, Shipman DW, Kram R, Kuo AD. Mechanical and metabolic requirements for active lateral stabilization in human walking. Journal of biomechanics. 2004;37(6):827–835. 10.1016/j.jbiomech.2003.06.002 15111070

[56] Hof A, Vermerris S, Gjaltema W. Balance responses to lateral perturbations in human treadmill walking. The Journal of experimental biology. 2010;213(15):2655–2664. 10.1242/jeb.042572 20639427

[57] Kuo AD. Stabilization of lateral motion in passive dynamic walking. The International journal of robotics research. 1999;18(9):917–930. 10.1177/02783649922066655

[58] MacKinnon CD, Winter DA. Control of whole body balance in the frontal plane during human walking. Journal of biomechanics. 1993;26(6):633–644. 10.1016/0021-9290(93)90027-C 8514809

[59] Carver SG, Cowan NJ, Guckenheimer JM. Lateral stability of the spring-mass hopper suggests a two-step control strategy for running. Chaos: An Interdisciplinary Journal of Nonlinear Science. 2009;19(2):026106 10.1063/1.3127577 19566266

[60] Bender CM, Orszag SA. Advanced Mathematical Methods for Scientists and Engineers I. Springer Science & Business Media; 1999.

